# Infiltrative Cutaneous Hemangiolipoma in a Goat

**DOI:** 10.1155/2013/942351

**Published:** 2013-07-11

**Authors:** Jessica R. Collier, Stacey R. Byers, Paula A. Schaffer, Deanna R. Worley, E. J. Ehrhart, Colleen G. Duncan, Alicia N. Grossman, Timothy Holt, Robert J. Callan

**Affiliations:** ^1^Department of Clinical Sciences, College of Veterinary Medicine and Biological Sciences, Colorado State University, Fort Collins, CO 80523-1620, USA; ^2^Flint Animal Cancer Center, Colorado State University, Fort Collins, CO 80523, USA; ^3^Department of Microbiology, Immunology, and Pathology, College of Veterinary Medicine and Biological Sciences, Colorado State University, Fort Collins, CO 80523, USA; ^4^Gunnison Valley Veterinary Clinic, Gunnison, CO 81230, USA

## Abstract

An approximately 4-year-old castrated male, Saanen cross goat presented to the Colorado State University Veterinary Teaching Hospital for evaluation and removal of a 22 cm × 22 cm, dark red, thickened, and crusted cutaneous lesion along the left ventrolateral thorax. An initial incisional biopsy performed approximately 8 weeks earlier was suspicious for cutaneous hemangiosarcoma. Surgical excision was deemed to be the most appropriate treatment option for this goat. A complete physical exam, complete blood count, and chemistry profile were performed and results were within normal limits. Thoracic radiographs and abdominal ultrasound were performed to rule out metastatic disease and comorbid conditions; no metastatic lesions or other abnormalities were observed. En bloc surgical excision of the affected skin was performed and the entire tissue was submitted for histopathology. A final diagnosis of cutaneous hemangiolipoma was reached upon extensive sectioning and histologic examination of the larger tissue specimen. The goat recovered well from surgery and has had no further complications up to 9 months postoperatively. To our knowledge, this is the first case report of a hemangiolipoma in a goat and surgical excision for such lesions appears to be a viable treatment method.

## 1. Introduction

Angiolipomas are rare tumors of endothelial cells that have been documented in both humans and animals [1–3]. These lesions are usually circumscribed masses of neoplastic endothelial cells that form well-differentiated vascular spaces and are supported by abundant adipose tissue. Angiolipomas may be categorized into hemangiolipomas or lymphangiolipomas based on whether or not the proliferating vascular spaces contain blood cells or lymph fluid. In humans, hemangiolipomas have been reported in the subcutis of the thorax, spine, gastrointestinal tract, and bone [4–6]. In dogs, individual case reports exist of angiolipomas arising in skin, bone of the spinal canal and parotid salivary gland [3, 7, 8]. A pericardial hemangiolipoma was reported in one bull [9]. Cutaneous hemangiolipomas have not been previously reported in goats. 

## 2. Material and Methods

An approximately 4-year-old, 100 kg castrated male, Saanen cross goat presented to the Colorado State University Veterinary Teaching Hospital (VTH) for evaluation and removal of a large cutaneous lesion along the left ventrolateral thorax. The lesion was first noted 1.5 years earlier and was thought to have started as irritation from a pack saddle. The lesion was not pruritic or painful; however, its location interfered with placement of the pack saddle. The referring veterinarian had performed an incisional biopsy 8 weeks prior to presentation at the VTH. Histopathology of the tissue was highly suspicious for a well differentiated hemangiosarcoma. 

**Figure 1:**
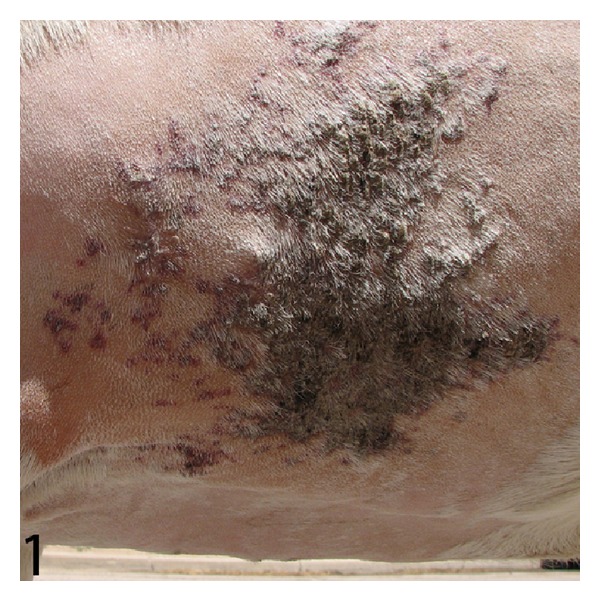
Goat. Gross appearance of the cutaneous mass in situ. Cranial is to the left.

On physical examination an approximately 22 × 22 cm, fairly discrete region of skin caudal to the left axilla was palpably thickened (approximately 1 cm thick) and firm, mildly alopecic, discolored red and purple, and densely crusted with dark red scabs (Figure 1). The remainder of the physical exam was within normal limits (temperature 39°C, pulse 60 beats per minute, respiration 50 breaths per minute, 2 rumen contractions per minute, and body condition score 6/9). Presurgical blood work was within normal limits for goats. Thoracic radiographs and abdominal ultrasound were unremarkable. 

An intravenous catheter was placed in the left jugular vein and the patient was prepared for general anesthesia and surgery. Sedation was achieved using hydromorphone (0.025 mg/kg, IV) and diazepam (0.05 mg/kg, IV). Induction was performed with ketamine (4 mg/kg, IV), diazepam (0.15 mg/kg, IV), and propofol (0.3 mg/kg, IV). The patient was intubated and maintained on isoflurane during surgical excision of the mass. Surgical margins were drawn within 1 cm of the lesion in an elliptical shape approximately 25 × 35 cm, originating at the left axilla and extending dorsally along the left thoracic wall. Monopolar electrocautery was used to incise the skin along the margins. The skin incision was extended just deep to the panniculus muscle, and the flap of lesional tissue was dissected medial to this tissue plane. During dissection, mild areas of subcutaneous and dermal bruising were evident but did not extend into tissues deeper than the panniculus. Temporary margin apposition was achieved as the flap was dissected using large towel clamps. Once the lesion was completely excised, surgical closure began with tension-relieving cruciate and interspersed buried simple interrupted sutures using 0 monofilament polyglyconate (Maxon, Covidien, Mansfield, MA, USA) within the panniculus muscle fascia at either end of the ellipse. Once apposition of the skin was achieved, an intradermal horizontal mattress suture pattern was placed using 2-0 monofilament polyglyconate. A Ford interlocking pattern using 0 polybutester (Novafil, Covidien, Mansfield, MA, USA) was placed over the intradermal pattern. The resulting apposed incision was approximately 45 cm in length. Finally, 1 polybutester was used to place 12 tie-over bandage loops surrounding the incision. Four lap sponges were rolled and secured over the incision using umbilical tape. Perioperative medications included tulathromycin (2.5 mg/kg, SC [Draxxin, Zoetis, Madison, NJ, USA]), flunixin meglumine (1.1 mg/kg, IV, q 12 hrs), and morphine (0.09 mg/kg, SC, q 8 hrs). The goat recovered well after surgery and anesthesia and returned home several days later on a course of oral meloxicam (1 mg/kg, PO, q 24 hrs) for pain management. An incisional seroma developed at the most ventral portion of the incision approximately 1 week after surgery. The ventral-most sutures were removed to allow for drainage and the incision healed without further complication. Remaining sutures were removed 3 weeks after surgery. 

The entire cutaneous lesion was fixed for >24 hrs in 10% neutral buffered formalin. Representative tissues were trimmed, embedded in paraffin, sectioned at 4 *μ*m, and stained with hematoxylin and eosin for histological evaluation. 

## 3. Results

**Figure 2:**
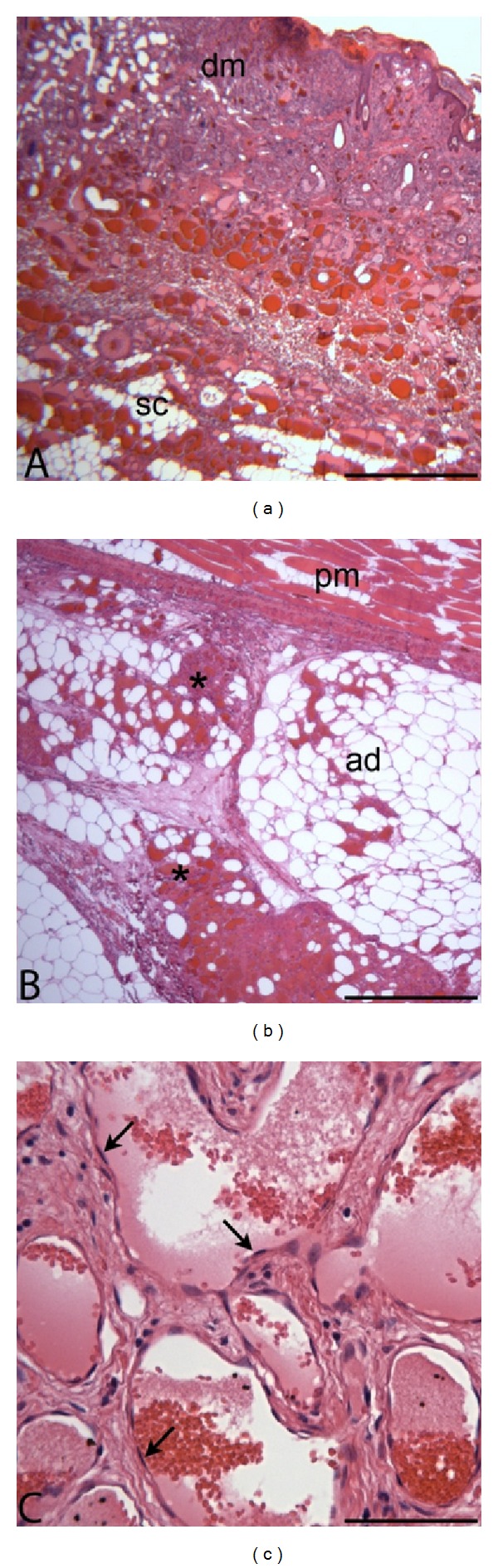
(a) Photomicrograph, goat, cutaneous mass. Subgross image of biopsied skin and panniculus tissue demonstrates neoplastic endothelial cells lining vascular channels in the dermis (dm) and subcutis (sc). H&E stain; bar = 250 *μ*m. (b) Photomicrograph, goat, cutaneous mass. High power image of biopsied tissue. Neoplastic endothelial cells (asterisks) are present deep to the panniculus muscle (pm) and infiltrating amongst lightly encapsulated lobules of mature adipose tissue (ad). H&E stain; bar = 250 *μ*m. (c) Photomicrograph, goat, cutaneous mass. Neoplastic endothelial cells (arrows) are flattened with minimal atypical features. H&E stain; bar = 100 *μ*m.

Large numbers of neoplastic endothelial cells formed an expansile, poorly demarcated, and highly infiltrative mass in the superficial dermis, deep adipose, and subjacent skeletal muscle. The neoplastic cells were arranged in delicate vascular channels (50 to 80 *μ*m in diameter) supported by dermal collagen (Figure 2(a)) or by lobules of well-differentiated adipocytes (Figure 2(b)). The endothelial cells were spindle shaped with scant eosinophilic cytoplasm and flattened to minimally bulging nuclei with dense chromatin (Figure 2(c)). There was negligible anisocytosis and anisokaryosis, and mitotic figures were rare (<1 per 10 high power field, 400x). The neoplastic endothelial cells penetrated the panniculus muscle and intercalated amongst lightly encapsulated lobules of well-differentiated adipose. Where tumor cells infiltrated muscle there were mild multifocal myocyte degeneration and atrophy. Adipocytes amongst the tumor cells were moderately variable in size and shape but lacked features of atypia. The overlying epidermis was multifocally eroded and ulcerated, with accumulations of dense serocellular crusts that were interpreted as secondary to trauma. Neoplastic cells extended to the side margins of the tissue submitted. No actinic changes were identified in the sections of dermis or epidermis examined. Taken together, the histological features were compatible with hemangiolipoma.

## 4. Discussion

In dogs and cats, angiolipomas are typically small discrete masses located in the subcutis of the thorax [3]. In humans favored sites include both the thorax and extremities [10]. Infiltrative angiolipomas in humans may have a predisposition for localization in the neck [11]. Angiolipomas are defined histologically by neoplastic blood vessels mixed with mature adipose tissue [2, 3]. Mitotic figures are rare or absent in both the endothelial and adipocyte population, and features of malignancy are lacking. Fibrin thrombi may be evident within vascular channels. 

The angiolipoma described in this case was located on the thorax and was comprised of thin walled vascular channels lined by well differentiated endothelial cells and supported by lobules of well-differentiated adipose. Unlike angiolipomas described in other species, which are typically discretely marginated, the mass in this case invaded the deep cutaneous musculature, resulting in mild myocyte degeneration and loss. Importantly, invasive angiolipomas may be difficult to distinguish from intramuscular hemangiomas, which in humans and other species are represented by primary development of a cavernous vascular neoplasm within skeletal muscle, often accompanied by abundant adipose tissue [12]. In this case, the majority of the mass effect was restricted to the dermis and subcutis and the lesion was interpreted as secondarily invading adjacent muscle, rather than originating within it.

Cutaneous neoplasms are uncommon in goats in general. Reported cutaneous neoplasms in goats include squamous cell carcinoma, squamous papilloma, epithelioma, apocrine sweat gland adenoma, fibroma, melanoma, mast cell tumor, histiocytoma, hemangioma, and hemangiosarcoma [10, 13, 14]. As in other species, factors such as breed, genetics, coat and skin color, and environment likely contribute to oncogenesis in caprine skin. Unfortunately, specific oncogenic factors have not been well studied in goats. As reported in other species, caprine cutaneous tumors often arise in lightly pigmented breeds or in areas of white or lightly pigmented hair, suggesting that UV radiation may play a role in oncogenesis [15–18]. Compatible with this predisposition, the patient in this report was lightly colored, though the direct relationship between UV exposure and the lesion in this case is uncertain, since actinic changes were not identified in the adjacent or marginal skin tissue. Also intriguing is the patient's history as a pack animal and the location of the lesion in the pack saddle area. Chronic inflammation has also been associated with development of cancer in several species, presumably due to the long-term effects of locally produced cytokines and growth factors intended to stimulate growth for healing purposes. For example, chronic corneal inflammation can lead to secondary corneal neoplasia in dogs, and chronic inflammatory bowel disease in cats is a well-recognized risk factor for intestinal lymphoma [19, 20]. Additionally, cutaneous papillomas and squamous cell carcinomas have been reported to develop in cattle at sites of traumatic freeze and heat branding [21, 22]. The tumor in the patient reported here may have arisen under the influences of chronic irritation and inflammation associated with tack.

Other differentials considered for nonpruritic crusting lesions in small ruminants include hyperkeratosis secondary to trauma, dermatophilosis, staphylococcal dermatitis, zinc deficiency, pemphigus foliaceus, and ringworm (*Trichophyton verrucosum* and *Epidermophyton floccosum*) [15, 23]. Close evaluation of the biopsied material allowed rejection of these inflammatory conditions. Squamous cell carcinoma was considered based on gross appearance but was not supported by the histological findings. Hemangioma was considered because of the benign appearance of the endothelial cells, and hemangiosarcoma was considered because of the infiltrative nature of the lesion. Ultimately, infiltrative hemangiolipoma was considered the most appropriate diagnosis because of the relative monotony of the neoplastic population, lack of atypical features, low mitotic rate, invasion of deep muscle, and close association of neoplastic vessels with mature lobules of adipose tissue. 

Human and canine angiolipomas are considered benign neoplasms and surgical excision is curative [3]. Minimal data is available for this tumor type in other species, but benignancy is likely a consistent feature. The biologic behavior of this lesion in a goat is uncertain, but the degree of invasiveness suggests that clinical followup is warranted and that local recurrence may occur, particularly given incomplete excision. 

## 5. Conclusions

The patient was allowed to return to normal activity following suture removal. Recurrence of the cutaneous lesion at the surgery site was not identified at a 10-month followup. Surgical excision appears to be a viable treatment method for cutaneous hemangiolipomas in goats; however long-term patient monitoring is recommended due to the lack of clinically described cases. Importantly, the initial incisional biopsy was reported as consistent with hemangiosarcoma, a significantly more serious disease with a median survival time of 170–300 days [24]. Because of the potential risk of misdiagnosing vascular cutaneous tumors in goats, en bloc tumor excision followed by thorough histological examination is recommended for large caprine cutaneous lesions.
